# Evaluation of objective visual quality in dry eye disease and corneal nerve changes

**DOI:** 10.1007/s10792-020-01483-5

**Published:** 2020-07-02

**Authors:** Jiahui Ma, Shanshan Wei, Xiaodan Jiang, Yilin Chou, Yuexin Wang, Ran Hao, Jiarui Yang, Xuemin Li

**Affiliations:** Department of Ophthalmology, Beijing Key Laboratory of Restoration of Damaged Ocular Nerve, Peking University Third Hospital, Beijing, 100191 China

**Keywords:** Dry eye diseases, Objective visual quality, OQAS II, In vivo confocal microscope

## Abstract

**Purpose:**

To explore objective visual quality in dry eye diseases (DED) and the correlation between corneal nerves and objective visual quality.

**Methods:**

Ninety-eight eyes of 49 patients with DED were included. Each patient was evaluated with the ocular surface disease index (OSDI), eyelid margin signs and meibomian gland assessments; corneal staining; tear film breakup time (TBUT); tear meniscus height (TMH); in vivo confocal microscopic (IVCM); objective visual quality including the objective scatter index (OSI), mean objective scattering index (mOSI), modulation transfer function (MTF) cutoff value and Strehl ratio.

**Results:**

A significant correlation was found between the OSDI and mOSI (*r* = 0.422, *p* = 0.005), MTF cutoff value (*r* = − 0.355, *p* = 0.020), and Strehl ratio (*r* = − 0.446, *p* = 0.003). The OSI was significantly correlated with TBUTf (*r* = − 0.213, *p* = 0.042). The mOSI, MTF cutoff value, Strehl ratio were correlated with eyelid margin signs and meibomian assessments. Additionally, there was a statistically significant correlation between corneal nerve length and the mOSI (*r* = − 0.239, *p* = 0.037), OSI (*r* = − 0.294, *p* = 0.028), MTF cutoff value(*r* = 0.282, *p* = 0.012), and Strehl ratio (*r* = 0.299, *p* = 0.008).

**Conclusions:**

Our study explored that objective visual quality was correlated with clinical symptoms and signs in DED patients. Furthermore, for the first time, our study explored the relationship between corneal nerves and objective visual quality and discovered that longer and wider corneal nerves were associated with better objective visual quality, which suggested that nerve changes may be a factor that related to poor visual quality in DED patients.

## Introduction

DED is a multifactorial disease of the ocular surface characterized by a loss of homeostasis of the tear film, associated with ocular symptoms. Tear film instability, ocular surface inflammation and neurosensory abnormalities all can be etiological factors in DED [1]. This chronic ocular surface disease can cause decreased visual quality and various complications that may even lead to a reduction in quality of life [2]. Although visual acuity is often normal by standard scales, visual quality may be poor due to the impact of DED. In patients with severe DED, visual quality can be permanently damaged, but the correlation between DED and visual quality remains unclear. Most pathogeneses refer that poor visual quality is associated with unstable tear film, and several studies have reported that tear film is a vital component of clear vision [3–6]. Rieger et al. [7] first underscored the significance of intact, stable precorneal tear film for good visual quality in 1992. Tan et al. showed that DED had significant alterations of visual quality compared to the control subject, the instability of the tear film directly decreases the quality of vision. Koh et al. [3] discovered that intraocular scattering and corneal backward scattering were higher in a DED group.

More recently, the optical quality analysis (OQAS) was widely used to assess objective visual quality. The OQAS is an instrument based on double-pass (DP) technology, which can obtain the visual quality of the eyes based on aberration, scattering and diffraction objectively and dynamically. The first pathway is from a laser source to the retina. The second pathway is from the retina to a charge-coupled device camera. This method has been used in cataract and refractive surgery. In recent years, the findings can also be used for the diagnosis of dry eye disease (DED).

There are some studies regarding the changes in the corneal nerves among DED patients, and these studies observed a significant decrease in corneal nerve length or density in DED patients [8–13]. A few studies demonstrated that DED patients may have abnormal morphology, such as abnormal nerve tortuosity and reflectivity [8, 12, 13]. We hypothesize that corneal damage induces aberrations and scattering that result in decreased visual quality. Wang et al. [14] reported that corneal nerve fiber density was positively correlated with subjective visual quality. However, there are no reports about the correlation between objective visual quality and the corneal nerve. To explore and evaluate changes in visual quality in DED and its pathogenesis, we performed this study to analyze the objective visual quality and various clinical signs of DED patients. Objective visual quality and the corneal nerve were first quantitatively analyzed in this study, and we hypothesized that objective visual quality and corneal nerve damage have strong correlations in DED.

## Materials and methods

### Patients

This prospective study included 98 eyes of 49 patients with DED who were recruited from the Department of Ophthalmology in Peking University Third Hospital from December 2017 to October 2018. Inclusion criteria included the following: positive symptomatology including sensitivity to light, foreign body sensation, burning, and blurred vision for more than 3 months; plus noninvasive BUT < 10 s or positive ocular surface staining [1]. Exclusion criteria included the following: (1) aged < 18 years, (2) any history of wearing contact lenses within the past 3 months, (3) best-corrected visual acuity (BCVA) < 20/20, (4) recent ocular surgery within 3 months and (5) history of trauma or other ocular diseases.

This study was conducted following the tenets of the Declaration of Helsinki. Written informed consent was obtained from all participants after explanation of the nature and possible consequences of the study. The protocol was approved by the local review board.

### Measurements

The clinical assessments of the enrolled subjects were conducted in the following order: firstly best-corrected visual acuity(BCVA), ocular surface disease index (OSDI), objective visual quality, tear meniscus height (TMH), tear breakup time (TBUT), eyelid margin signs and meibomian gland assessments, then corneal staining and in vivo confocal microscopy (IVCM). An interval of 5 min was required between different tests.

### Ocular surface disease index (OSDI)

Dry eye symptoms were measured by the OSDI, which also measured environmental triggers and quality of life associated with vision. The OSDI was quantified on a scale from 0 to 100. Higher scores indicated more severe symptoms [1].

###  OQAS II

The patients were assessed by an observer who was blinded to the details of clinical observation using OQAS II (Visiometrics S.L., Tarrasa, Spain). For all the subjects, the DP images were acquired at the best focus by using an optometer to correct internally refractive error if necessary. In order to reach the maximum possible natural pupil size, the DP image was obtained after a period of dark adaptation. The patient’s head was positioned on a chin rest and stared at the center of a figure. Subjects were asked to blink before the measurement until the DP recorder began to register.

**Fig. 1:**
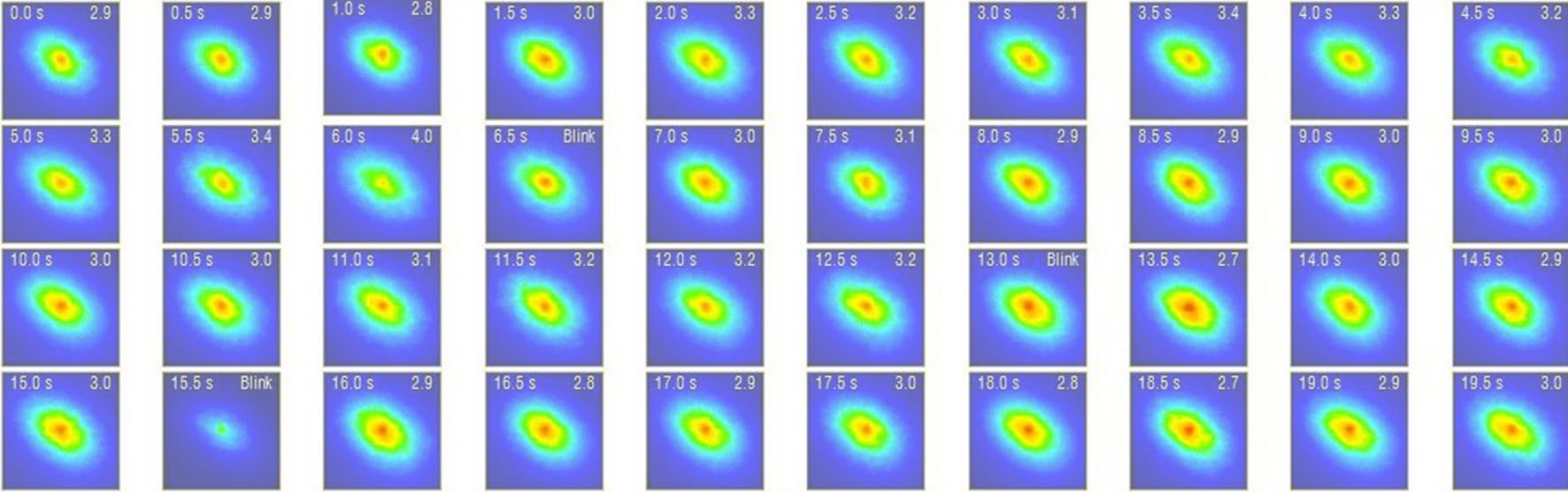
Example of tear film analysis in the OQAS II. The OSI was recorded every 0.5 seconds within a 20-second period

From each image, we had several main parameters. The first parameter was the objective scattering index (OSI), which is a measure of the amount of light that is scattered as it passes through the ocular structure, which is defined as the ratio of intensity at an eccentric location to the central area in a DP image [15]. The OSI was measured just after the patient blinked, and we recorded the OSI every 0.5 s within a 20-second period, which is shown in Fig. 1. Higher OSI value indicated a higher level of intraocular scattering. The mean OSI is the mean objective scattering index of tear film. The second measurement was the modulation transfer function (MTF). The ordinate axis is the contrast ratio between the object and image, and the horizontal axis corresponds to the spatial frequencies. The MTF curve represents the contrast attenuation percentage of retinal images at different frequencies, considering all the optical defects involved in retinal imaging, including the effects of scattering and high degree optical aberrations. The intersection of the MTF curve and the abscissa is the cutoff frequency, reported as MTF cutoff value. The third measurement was the Strehl ratio, which is calculated as the ratio between the area under the MTF curve and that of an aberration-free eye [15]. A ratio closer to 1 indicates a smaller aberration of the eye.

#### Tear meniscus height (TMH) and tear breakup time (TBUT)

TMH and TBUT were assessed by a Keratograph 5 M (Oculus, Wetzlar, Germany). TMH is defined as the length of a vertical line from the top of the inferior tear meniscus to the eyelid margin. It was measured and recorded using Oculus. TBUT is the interval of time that elapses after having a complete blink and the appearance of the first break, noted as TBUTf. Firstly, we introduced the method of examination briefly. The patients closed eyes for a while, and then, they looked at a red target in the front, adjusted the focal length of the instrument. The system would automatically prompt patients to blink twice and keep their eyes open as long as possible [16].

#### Eyelid margin signs and Meibomian gland assessments

The anterior eye was examined with a slit-lamp biomicroscope, and eight signs of the eyelid margin and meibomian gland were assessed in our study as follows: rounding of the posterior margin, irregularity of the margin, lash loss, trichiasis, blepharitis, vascularity of the lid margin or telangiectasia, plugging of orifices in the upper or lower lid, and meibum quality. The degree of meibomian gland deficiency was the ratio of the total meibomian gland area to the total meibomian area, which can be divided into 4 grades: 1 is normal, 2 is more than 2/3, 3 is 1/3–2/3, 4 is less than 1/3. Meibum quality was assessed in each gland using a scale of 0–3 for each gland as follows: 0, clear; 1, cloudy; 2, cloudy with debris or granular; and 3, thick like toothpaste, and was recorded as the highest grade expressed by all examined glands. The assessment was performed according to the previous reports [17].

#### Corneal staining

Corneal staining was assessed using corneal NaFl. Optimal viewing ranges between 1 and 3 min after instillation under the slit-lamp using cobalt blue illumination. We made two perpendicular lines in the center of the cornea and divided the cornea into four parts. Staining was graded from 0 to 3 in each of the 4 sections. The total score ranged from 0 to 12.

#### In vivo confocal microscopy

IVCM (Heidelberg Engineering, Heidelberg, Germany) has lately been used to evaluate nerve alterations in patients with DED because it is the only method by which we could take an image of the subbasal nerve plexus. IVCM is a noninvasive imaging modality that enables us to study the living cornea at the cellular level rapidly with good repeatability. With the combination of a numerical aperture and an objective lens (X63/0.9), we could get a high magnification of image up to 800-fold. IVCM uses a 670-nm wavelength helium–neon diode laser and scans the examined field in a raster pattern. A drop of oxybuprocaine was administered into the conjunctiva sac for topical anesthesia before the examination. The patients were asked to place their chin and forehead on the rest and were required to gaze at a certain fixation. Ophthalmic gel was filled into the objective lens. The examiner adjusted the location and focus of the lens to obtain a clear image. All subjects were asked to keep still throughout the entire scan time to ensure that images could be captured from the corneal apex. The overall examination took approximately 5 min for one subject. Images captured by the IVCM have a definition of 384 × 384 pixels over an area of 400 µm × 400 µm, with a lateral spatial resolution of 0.5 µm and a depth resolution of 1–2 µm. All the images were obtained used the “volume scan” setting, which captured 30 to 40 images for each eye, from the corneal epithelium to the endothelium. For each cornea, images were selected from the subbasal layer, lying parallel to the corneal surface, between the corneal basal epithelium and Bowman’s layer, which has the richest plexus. There were about 3–5 images of the subbasal layer. We excluded the images with a strong artifact, and they should have at least one clearly visible nerve. Three images with optimal quality were selected for nerve analysis. A semiautomatic qualification image processing software (ImageJ, National Institutes of Health, Bethesda, MD) and a plug-in (NeuronJ, Biomedical Imaging Group, Lausanne, Switzerland) was applied to trace and quantify the structures of nerve. Length, width, reflectivity and tortuosity of the subbasal nerves was calculated following the tracing. Data were recorded as the mean values of 3 measurements.

#### Statistical analysis

All statistical tests were performed using SPSS 20.0 (SPSS, Inc., Chicago, IL, USA). The association between variables was examined using Spearman’s correlation analysis. All P values were considered significant when the value was less than 0.05.

## Results

Ninety-eight eyes of 49 DED patients who met the inclusion criteria were included in our study. The mean age of the patients was 56 ± 17 years (range 25–86 years). All patients completed the information collection process and examinations, and the results are shown in Table 1.

**Table 1:** Basic characteristics of patients and examination results

	Mean ± SD (range)
Gender	14 males/25 females
Age (years)	56 ± 17 (25–86)
OSDI	36.6 ± 17.3 (2.5–68.18)
OQAS	
OSI	1.40 ± 1.70 (0.2–11.00)
mOSI	2.00 ± 1.85 (0.42–11.28)
MTF cutoff value	32.3 ± 11.3 (3.5–55.1)
Strehl ratio	0.16 ± 0.05 (0.05–0.30)
TMH, mm	0.21 ± 0.06 (0.10–0.37)
TBUTf, s	5.9 ± 3.3 (1.7–20.8)
Eyelid margin, *n* (%)	
Rounding of posterior margin	33 (33.7)
Irregularity	31 (31.6)
Telangiectasia	37 (37.8)
Blepharitis	13 (13.3)
Trichiasis	6 (6.1)
Lashloss	2 (2.0)
Meibomian gland assessments	
Meibum quality	1.73
Meibomian glands plugging	8 (8.2)
Degree of meibomian gland deficiency	2.86
Corneal staining	1.07
Confocal	
Corneal nerve length (pixels)	3862.3 ± 761.9 (2097.8–5807.1)
Corneal nerve width (pixels)	5.1 ± 0.8 (3.1–7.4)
Corneal nerve reflectivity (gray values)	137.3 ± 17.4 (95.0–170.6)
Corneal nerve tortuosity (degrees)	128.9 ± 6.9 (111.3–141.6)

*OSDI* ocular surface disease index, *TBUTf* first tear breakup time, *TMH* tear meniscus time, *OQAS* optical quality analysis system, *OSI* objective scattering index, *mOSI* mean objective scattering index, *MTF cutoff value* modulation transfer function cutoff

### Clinical symptoms

Considering clinical symptoms, the OSDI was significantly correlated with the mOSI (*r* = 0.422, *p* = 0.005), MTF cutoff value(*r* = − 0.355, *p* = 0.020), and Strehl ratio (*r* = − 0.446, *p* = 0.003). Table 2 shows the correlation between DED symptoms and OQAS parameters. Based on these results, there was a negative correlation between symptoms and objective quality.

**Table 2:** Results of correlations between eyelid margin signs and OQAS parameters

Variable	OSDI	Rounding	Notching	Vascularity	Trichiasis	Plugging	Lass loss	Blepharitis	Meibum quality
mOSI									
*r*	0.422	0.223	0.077	0.203	0.041	0.306	0.222	0.240	0.214
*p*	0.005*	0.037*	0.477	0.057	0.706	0.004**	0.038*	0.025*	0.048*
OSI									
*r*	0.297	0.293	0.147	0.279	0.041	0.306	0.228	0.255	0.330
*p*	0.053	0.005**	0.170	0.008*	0.702	0.004**	0.032*	0.016*	0.02*
MTF cutoff value									
*r*	− 0.355	− 0.324	− 0.069	− 0.206	− 0.084	− 0.225*	− 0.200	− 0.186	− 0.298
*p*	0.020*	0.002**	0.518	0.051	0.431	0.033*	0.058	0.080	0.005**
Strehl ratio									
*r*	− 0.446	− 0.294	− 0.132	− 0.278	− 0.092	− 0.264	− 0.221	− 0.239	− 0.195
*p*	0.003**	0.005**	0.216	0.008**	0.390	0.012*	0.037*	0.023*	0.069

**Significant correlation (*p* < 0.01)

*Significant correlation (*p* < 0.05)

### Eyelid margin signs and meibomian gland assessments

A significant relationship was detected between the mOSI and eyelid margin rounding (*r* = 0.223, *p* = 0.037), plugging (*r* = 0.306, *p* = 0.004), lash loss (*r* = 0.222, *p* = 0.038), blepharitis (*r* = 0.240, *p* = 0.025), and meibum quality (*r* = 0.214, *p* = 0.048). The MTF cutoff value was significantly correlated with rounding (*r* = − 0.324, *p* = 0.002), plugging (*r* = − 0.225, *p* = 0.033), and meibum quality (*r* = − 0.298, *p* = 0.005). The Strehl ratio was significantly correlated with rounding (*r* = − 0.294, *p* = 0.005), vascularity (*r* = − 0.278, *p* = 0.008), plugging (*r* = − 0.264, *p* = 0.012), lash loss (*r* = − 0.221, *p* = 0.037), and blepharitis (*r* = − 0.239, *p* = 0.023). Table 2 shows the results of correlations between eyelid margin signs and OQAS parameters.

### Tear breakup time, Tear meniscus height and corneal staining

Concerning tear film clinical tests, TBUTf was significantly correlated with the OSI (*r* = − 0.251, *p* = 0.016), and MTF cutoff value(*r* = 0.213, *p* = 0.042), there was no correlation between TMH and objective visual quality. The correlations between tear film clinical tests and OQAS parameters are presented in Table 3. In brief, longer TBUTf was associated with more stable tear film and better objective visual quality.

**Table 3:** Results of correlations between tear film clinical tests and OQAS parameters

	TMH	TBUTf	Cornea Staining
mOSI			
*r*	− 0.056	− 0.036	0.029
*p*	0.601	0.740	0.855
OSI			
*r*	− 0.010	− 0.251	0.028
*p*	0.926	0.016*	0.860
MTF cutoff value			
*r*	− 0.014	0.213	− 0.029
*p*	0.892	0.042*	0.855
Strehl ratio			
*r*	− 0.017	0.120	− 0.086
*p*	0.875	0.256	0.581

**Significant correlation (*p* < 0.01)

*Significant correlation (*p* < 0.05)

### In vivo confocal microscopy

**Fig. 2:**
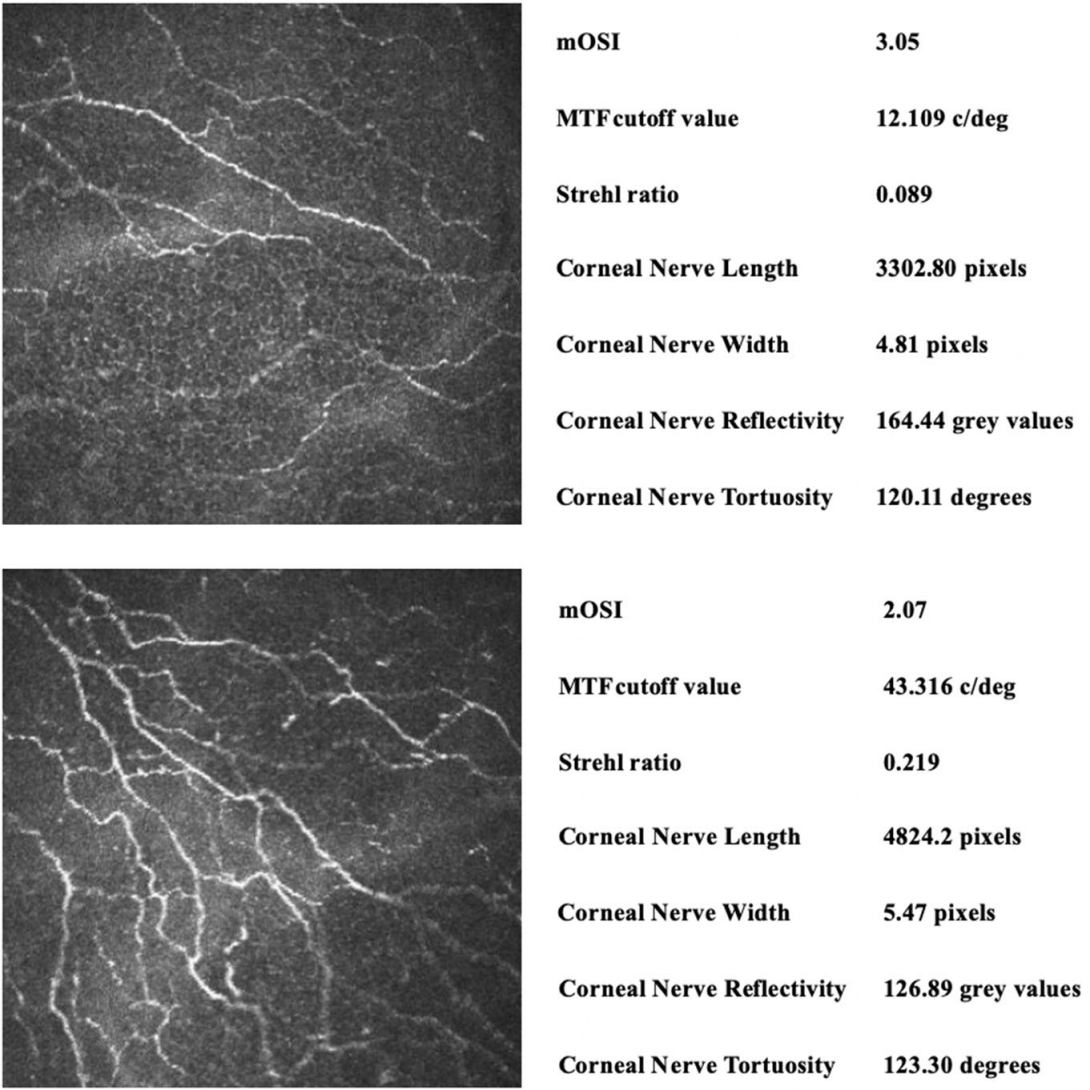
Examples of confocal image of corneal nerve with different OQAS parameters. Longer and wider corneal nerves were associated with better objective visual quality. c/deg, cycles per degree

Table 4 shows the results of correlations between IVCM and OQAS. There was a statistically significant correlation between corneal nerve length and all of the parameters in the OQAS, including the mOSI (*r* = − 0.239, *p* = 0.037), OSI (*r* = − 0.294, *p* = 0.028), MTF cutoff value (*r* = 0.282, *p* = 0.012), and Strehl ratio (*p* = 0.299, *r* = 0.008). Thus, longer nerve length indicated better vision quality. Additionally, corneal nerve width was correlated with the OSI (*r* = − 0.282, *p* = 0.015), MTF cutoff value (*r* = 0.259, *p* = 0.025), and Strehl ratio(*r* = 0.262, *p* = 0.023). Corneal nerve reflectivity and OSI were correlated (*r* = − 0.248, *p* = 0.028). By contrast, none of the corneal nerve parameters were correlated with corneal nerve tortuosity. Specifically, longer and wider corneal nerves were associated with better objective visual quality, as shown in Fig. 2.

**Table 4:** Results of correlation between in vivo confocal microscopy and OQAS parameters

	Corneal length (pixels)	Corneal width (pixels)	Corneal Reflectivity (gray values)	Corneal tortuosity (degrees)
mOSI				
*r*	− 0.239	− 0.180	0.139	− 0.056
*p*	0.037*	0.127	0.228	0.627
OSI				
*r*	− 0.294	− 0.180	− 0.282	− 0.56
*p*	0.009**	0.127	0.015*	0.629
MTF cutoff value				
*r*	0.282	0.259	− 0.094	0.007
*p*	0.012*	0.025*	0.411	0.951
Strehl ratio				
*r*	0.299	0.262	− 0.095	0.058
*p*	0.008**	0.023*	0.404	0.612

*Significant correlation (*p* < 0.05)

**Significant correlation (*p* < 0.01)

## Discussion

Our study explored a direct correlation between objective visual quality and clinical symptoms and signs in DED patients. Furthermore, to our best knowledge, the relationship between corneal nerve and objective visual quality was analyzed for the first time, and we determined that patients with longer and wider corneal nerves have better objective visual quality, which suggested that nerve changes and objective visual quality has a strong correlationship.

The OSDI is a scale for the assessment of symptoms related to DED and its effects on vision. The OSDI, one of the most commonly used questionnaires to measure the symptoms of DED, contains 3 parts, including ocular symptoms, environmental triggers, and quality of life associated with vision. More importantly, the OSDI evaluates patients’ vision-related quality of life. In our study, the OSDI score was significantly correlated with the mOSI, MTF cutoff value and Strehl ratio, consistent with a previous study [18]. The results demonstrated that the subjective sensation of the patients was related to their objective visual quality and that worse vision-related function was associated with poorer objective visual quality. The objective visual quality measured by OQAS may reflect visual function to a degree.

Our study discovered a negative correlation between visual quality and eyelid margin signs, as well as the meibomian gland assessments. Rounding and irregularity of the eyelid margin indicate abnormal meibomian gland conditions. These abnormal morphological changes caused abnormal meibum. Our study showed that the meibum quality was negatively correlated with objective visual quality. Poor meibum quality has a great impact on tear film, which increases the light scattering on the anterior surface of the cornea and causes overall ocular scatter [19]. Furthermore, in our study, vascularity and blepharitis were negatively correlated with visual quality. We hypothesized that the increased scattering might be related to aggregated inflammatory cells and inflammatory cytokines. Inflammation plays an important role in corneal nerve regeneration, but excessive inflammation might lead to decreased visual quality even keratopathy. In other words, the condition of the meibomian gland and the objective visual quality are closely linked, which demonstrates the importance of analyzing eyelid margin signs when assessing objective vision quality in DED patients.

In our study, objective visual quality was closely associated with tear film conditions. For example, we detected a significant correlation between visual quality and TBUTf. Higher TBUTf value was associated with lower OSI value, which was consistent with other published studies [20]. Regarding objective visual quality, many previous studies have verified the OSI as a new objective optical method to evaluate the quality and stability of the tear film [5, 18, 20–22]. Tear film is the first part of the ocular surface through which light passes to the retina and it can be considered as compensation for the cornea; without this compensation, there may be a significant difference in the optical path of the eye’s wavefront [4, 23, 24]. Thus, worse tear film conditions resulted in higher OSI values. MTF provides information about tiny alternations in the retinal image provoked by changes in tear film. A positive correlation was detected between the TBUTf and MTF cutoff value in our study: a higher TBUTf is associated with a higher MTF cutoff value. The decrease in the MTF cutoff value is explained by aberrations and wavefront caused by tear film. TBUT is one of the most important diagnostic tests for DED. Using serial parameters in OQAS could be a potential application in combination with TBUT to investigate tear film.

Our study was the first to explore the quantitative relationship between corneal nerve status and objective visual quality. We determined that patients with longer and wider corneal nerves had better objective visual quality. The cornea is the most innervated tissue in the human body, with a nerve density of 300 to 600 times that of the skin [25]. Corneal nerves decrease in a variety of ocular conditions, including corneal infections, injuries and surgeries, and any trigeminal nerve damage [26]. In addition to these conditions, the length of corneal nerves is reported significantly decreased in DED patients [10]. The pathogenesis of how corneal nerve changes influence the visual quality may be as follows. First, corneal nerves can sense touch, pain, and temperature and play an important role in the blink reflex, wound healing and tear secretion [27]. Under normal circumstances, the corneal nerve terminals contain sensors that can monitor the integrity of the tear film, which is necessary for vision function [28]. The sensory nerve on the cornea deploys afferent stimulation signals to the brain, which then returns an efferent signal [29]. Impaired corneal sensitivity results in a decreased blink and lacrimal reflexes, which influence the stability of tear film and therefore affect visual quality. Second, corneal nerves can also interact with limbal stem cells, epithelium and inflammatory cells. In the presence of abnormal corneal nerve conditions, these interactions would be disturbed, leading to corneal epithelial defects and even corneal scarring [27], which results in poor visual quality. However, the corneal staining in our investigation didn’t have a significant result. We will analyze it in further studies. Third, corneal nerves can release various trophic factors, including nerve growth factor (NGF), neurotrophins-3 (NT-3) and glial cell line-derived neurotrophic factors (GDNF), which can increase the epithelial integrity and promote wound healing [30].

We analyzed the relationship between DED and objective visual quality. Our study determined that more severe meibomian gland conditions and unstable tear film resulted in poorer objective visual quality. In addition, longer and wider corneal nerves indicated better objective visual quality. The present research indicated that the parameters of visual quality determined by OQAS might be feasible in evaluating the severity of dry eye and corneal nerve changes. However, our study included the following limitations. (1) The first limitation is the lack of a control group. Since we did not include a control group, we cannot determine whether corneal nerve changes in patients with dry eyes are related to visual quality or whether corneal nerves themselves are related to visual quality. Further studies should be carried out with control groups or in various ophthalmic diseases to rule out the above influences. (2) Second, the role of inflammation in the ocular surface diseases is significant. However, we did not summarize the changes in Langerhans cells. Further studies should focus on Langerhans cells to explore the function of inflammatory cells in visual quality. (3) Third, our study was conducted on DED patients of Peking University Third Hospital and it might not reflect the general population. (4) Our study also lacks fundamental researches to confirm the pathophysiological mechanism to explain the relationship between corneal changes and visual quality. (5) Finally, the numbers of enrolled patients with DED were small. (6) We proved that the objective visual quality and corneal nerve damage have a strong correlationship; however, more studies are needed for further discussion on the relationship.

Our study explored a direct correlation between objective visual quality and clinical symptoms and signs in DED patients. Furthermore, to our knowledge, this study is the first to explore the relationship between corneal nerves and objective visual quality. Our study provides novel insights into the mechanism of poor visual quality and further understanding of the molecular and cellular changes that occur in DED patients is necessary.

## Conclusions

Our study analyzed the relationship between DED and objective visual quality. We determined that more severe meibomian gland conditions, and higher TBUT indicated poorer objective visual quality. In addition, to our knowledge, our study is the first to explore the relationship between the corneal nerve and objective visual quality. Longer and wider corneal nerves were associated with better objective visual quality, which suggested that the corneal nerve changes might be a factor related to the poor visual quality in DED patients.

## Data Availability

The data used to support the findings of this study are included within the article.
